# PlantMedOman: an online database for Oman's medicinal plants

**DOI:** 10.6026/973206300200314

**Published:** 2024-04-30

**Authors:** Smitha Sunil Kumaran Nair, Aqsa Bibi, Nabras Al-Mahrami, Piyusha Mahesh Satam, Alya Al Farsi, Adhra Al Mawali, Nallusamy Sivakumar

**Affiliations:** 1 Department of Computing and Electronics Engineering, Middle East College, Sultanate of Oman; 2 National Genetic Center, Ministry of Health, Sultanate of Oman; 3 Dean's Office, Middle East College, Sultanate of Oman; 4 Quality Assurance and Planning, German University of Technology (GUtech), Sultanate of Oman; 5 Department of Biology, Sultan Qaboos University, Sultanate of Oman

**Keywords:** PlantMedOman, ethnomedicine, online database, Sultanate of Oman

## Abstract

The Sultanate of Oman has a rich biodiversity, particularly in medicinal plants, and plays a crucial role in traditional healthcare practices. However, the wealth of knowledge about these plants is scattered across various literature, making it challenging for researchers, practitioners, and the public to access comprehensive information. Therefore, the availability of a centralized, user-friendly online database to catalog Oman's medicinal plants is of great importance. PlantMedOman presented here, which currently holds 186 records helps to enhance academic research, support drug discovery studies, promote the conservation of medicinal plants, and foster greater awareness of Oman's ethnomedicinal heritage.

## Background:

Sultanate of Oman, a land distinguished by its diverse ecosystems, is home to a variety of medicinal plants, integral to the region's ethnomedicinal practices and these plants have been pivotal in traditional healthcare practices [1]. This rich biodiversity reflects the ecological wealth of the region [2]. A multi-centric study undertaken in India has led to a database of 500 medicinal plants from the Western Ghats that provides exhaustive details on each plant, including its scientific data, medicinal uses, and available literature on its pharmacology and toxicology. The database highlights over 200 diseases, 11,000 vernacular names in seven languages, and 800+ photographs, spanning 22 districts across five states in the country [3]. Another study in India endeavors to construct a comprehensive database that spans the entire Indian subcontinent, cataloging medicinal plants along with their phytochemical compositions and geographic distributions. The database encapsulates a total of 6,959 distinct medicinal plants spread across 348 families, with a presence in all 28 states and 8 union territories of India. It organizes information into four main categories: traditional knowledge, geographical indications, phytochemicals, and cheminformatics. Through this study, a vast array of 27,440 unique phytochemicals associated with these plants has been meticulously compiled from diverse sources [4]. Brazil's medicinal plant life data, featuring a comprehensive collection of 2,078 entries, derived from literature and herbarium sources is crafted in a digital format named Ewé database [5]. A database system model has been designed to offer a standardized platform for the submission, preservation, and access of ethnomedicinal information. Utilizing object-oriented database technology, this model is adept not just at holding data and multimedia content like digital images, audio, and video, but also at representing the domain-specific knowledge related to plant-based remedies found in traditional medicinal systems. It integrates linguistic and semantic components to enhance its functionality [6]. The Program for Collaborative Research in the Pharmaceutical Sciences at the University of Illinois at Chicago's College of Pharmacy hosts what is likely the most extensive ethnopharmacological database, known as NAPRALERT which stands for NAtural PRoducts ALERT, this database is the foremost relational database globally, cataloging information from the world literature on the ethnomedical or traditional uses, chemistry, and pharmacology of extracts from plants, and biological activities however offers limited free searches of the database [7]. A relational database was developed using data collected from various historical documents, such as diaries, travel narratives, and medicinal plant treatises authored by explorers, botanists, and physicians who visited Campania, a region in southern Italy, over the past three centuries. Additionally, the database incorporates ethnobotanical practices mentioned in historical herbal compilations and texts from the Ancient and Medieval periods of the Mediterranean Region. It documents 1,672 distinct applications spanning medicinal, dietary, ceremonial, and veterinary uses for 474 species cataloged within the database [8]. Herbal medicines are the cornerstone of advanced traditional medical systems and have led to the development of several key pharmaceuticals still in use. TarNet, a meticulously curated database and platform, focuses on traditional medicinal plants and their natural compounds, featuring detailed bio-target information. This database compiles data on proteins influenced by or associated with medicinal plant components, as well as on protein-protein interactions (PPIs). TarNet offers comprehensive insights into the relationships between plants, compounds, and proteins, along with PPIs. Furthermore, it facilitates network analysis of biological pathways and protein-protein interactions related to particular diseases, serving as a valuable resource for researchers [9]. PeruNPDB details the compilation, refinement, and chemoinformatic analysis of the Peruvian Natural Products Database version, focusing on its chemical space coverage, physicochemical properties, and chemical diversity, encompassing 280 natural products available in Peru, a megadiverse country with diverse endemic plant species. This highlights the direct and indirect impacts of biodiversity conservation on human health [10]. The TCM Database@Taiwan compiles data from ancient Chinese medical texts and contemporary scientific studies, offering an extensive non-commercial repository of traditional Chinese medicine (TCM) information. This online database features over 20,000 pure compounds derived from 453 TCM ingredients [11]. A literature survey identified 143 species of ethnomedicinal plants across 61 families, which are used by local communities in Bangladesh for the treatment of diabetes [12]. Therefore, it is of interest to describe development and maintenance of comprehensive an online database PlantMedOmanfor Oman's medicinal plants.

## Materials and Methods:

## Data gathering:

The data collection process commenced with a comprehensive search strategy employing specific keywords such as "Omani Medicinal Plants" and "Ethnomedicine in Oman." This targeted approach yielded two relevant literature sources. All available data from these sources were meticulously gathered and compiled. To ensure data integrity, any redundant records were carefully eliminated. The column structure of the dataset was determined based on the headings present in [1]. In instances where certain data fields were unavailable in [2], the corresponding locations were populated with the phrase "Not available". This approach ensured a consistent and comprehensive data collection framework of PlantMedOman.

## Design framework:

**Figure 1:**
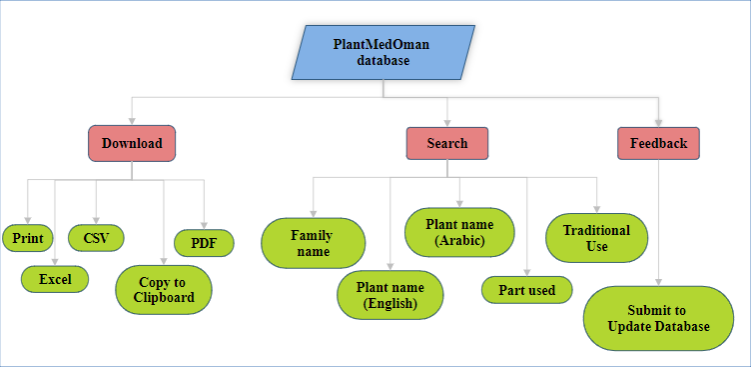
Workflow of PlantMedOman

The Oman Medicinal Plant website was created with Wordpress, a web content management system software. The prerequisites to creating and running a website online such as the domain and hosting were purchased from Omantel. All files, data, and contents associated with the website are stored and managed, making it accessible to users on the Internet. The WordPress dashboard which provides several themes, tools, and plugins has been utilized to install and customize to produce a user-friendly and responsive website. The theme used on our website is the Astra theme. The Elementor plugin is a widely popular page builder plugin that was used to create the appearance, layout, design, and add content to the website and publish it. wpDataTables is a Wordpress-based table plugin that allows for the creation and management of data tables and charts on websites. The plant species data was uploaded to the plugin in CSV format and advanced search and filters were added to the table to allow users to extract exact data according to their needs concerning the different table columns. Users are given the option to download the table data in diverse formats such as PDF, Excel, and CSV. In addition, options to copy and print the table data are enabled. The entire workflow with search, download, and feedback options is shown in Figure 1. The feedback form on the website is created and designed using the Contact Form 7 plugin. The mail sent through this form will by default be led to the spam folder because WordPress utilizes the PHP mail function. To prevent the emails from being marked as spam, the WP Mail SMTP plugin is installed which utilizes an external reliable SMTP server rather than the default PHP mail function.

## Utility:

**Figure 2:**
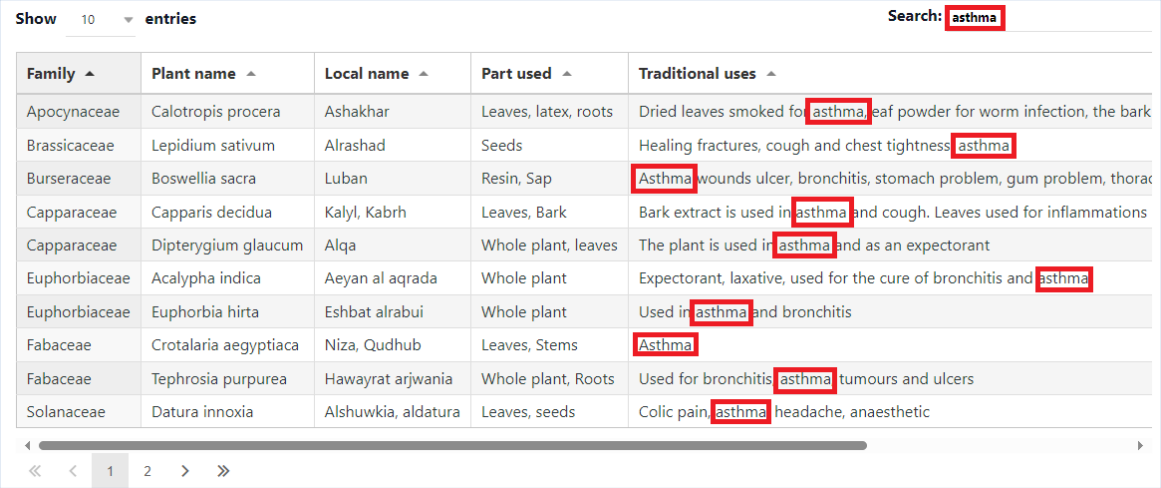
General search option in PlantMedOman, showing the records matching to a chosen keyword "asthma".

The web-based PlantMedOman database currently available at Oman Medicinal Plant (https://omanmedicinalplant.om/) comprises a total of 186 records that have been compiled to date. For all the database records, the column names are indicated by the family name, plant name, local name, parts used, and traditional uses in addition to the citation. A general search option available in PlantMedOman is depicted in Figure 2 showing the records matching the chosen keyword "asthma".

**Figure 3:**
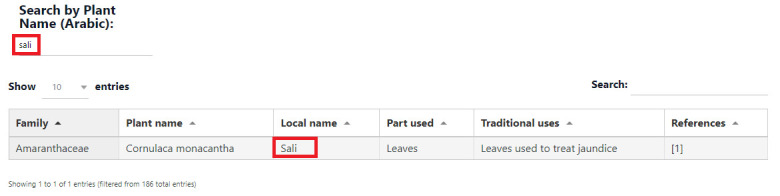
Search option by Plant Name (Arabic) in PlantMedOman, showing the records matching to the chosen name "Sali".

Users can download the data in multiple formats, including .csv and .xls, and have options to copy, print, and save. Additionally, PlantdbOman provides both straightforward and complex web-based search functionalities, allowing users to refine their searches based on specific criteria such as plant family, plant name in English or Arabic, part used, traditional usage, or disease name, according to the selected search choice and keywords. The keywords are non-case sensitive. A sample of a specific search option with results is depicted in Figure 3.

## Conclusion:

PlantMedOman helps to preserve invaluable traditional knowledge of Omani medicinal plants in a digital platform.
